# Discontinuation of antidepressant medication after mindfulness-based cognitive therapy for recurrent depression: randomised controlled non-inferiority trial

**DOI:** 10.1192/bjp.bp.115.168971

**Published:** 2016-04

**Authors:** Marloes J. Huijbers, Philip Spinhoven, Jan Spijker, Henricus G. Ruhé, Digna J. F. van Schaik, Patricia van Oppen, Willem A. Nolen, Johan Ormel, Willem Kuyken, Gert Jan van der Wilt, Marc B. J. Blom, Aart H. Schene, A. Rogier, T. Donders, Anne E. M. Speckens

**Affiliations:** **Marloes J. Huijbers**, MSc, Department of Psychiatry, Radboud University Medical Center, Nijmegen, The Netherlands; **Philip Spinhoven**, PhD, Institute of Psychology, Leiden University, Leiden and Department of Psychiatry, Leiden University Medical Center, Leiden, The Netherlands; **Jan Spijker**, PhD, MD, Pro Persona Nijmegen, Nijmegen, The Netherlands; **Henricus G. Ruhé**, PhD, MD, Department of Psychiatry, University Medical Center Groningen, Groningen, The Netherlands; **Digna J. F. van Schaik**, PhD, MD, **Patricia van Oppen**, PhD, GGZ InGeest and Department of Psychiatry, VU University Medical Center, Amsterdam, The Netherlands, **Willem A. Nolen**, PhD, MD, **Johan Ormel**, PhD, Department of Psychiatry, University Medical Center Groningen, Groningen, The Netherlands; **Willem Kuyken**, PhD, Department of Psychiatry, Warneford Hospital, University of Oxford, Oxford, UK; **Gert Jan van der Wilt**, PhD, Department for Health Evidence, Radboud University Medical Center, Nijmegen, The Netherlands; **Marc B. J. Blom**, PhD, MD, Parnassia Bavo Psychiatric Institute, The Hague, The Netherlands; **Aart H. Schene**, PhD, MD, Department of Psychiatry, Radboud University Medical Center, Nijmegen, The Netherlands; **A. Rogier T. Donders**, PhD, Department for Health Evidence, Radboud University Medical Center, Nijmegen, The Netherlands; **Anne E. M. Speckens**, PhD, MD, Department of Psychiatry, Radboud University Medical Center, Nijmegen, The Netherlands

## Abstract

**Background**

Mindfulness-based cognitive therapy (MBCT) and maintenance antidepressant medication (mADM) both reduce the risk of relapse in recurrent depression, but their combination has not been studied.

**Aims**

To investigate whether MBCT with discontinuation of mADM is non-inferior to MBCT+mADM.

**Method**

A multicentre randomised controlled non-inferiority trial (ClinicalTrials.gov: NCT00928980). Adults with recurrent depression in remission, using mADM for 6 months or longer (*n* = 249), were randomly allocated to either discontinue (*n* = 128) or continue (*n* = 121) mADM after MBCT. The primary outcome was depressive relapse/recurrence within 15 months. A confidence interval approach with a margin of 25% was used to test non-inferiority. Key secondary outcomes were time to relapse/recurrence and depression severity.

**Results**

The difference in relapse/recurrence rates exceeded the non-inferiority margin and time to relapse/recurrence was significantly shorter after discontinuation of mADM. There were only minor differences in depression severity.

**Conclusions**

Our findings suggest an increased risk of relapse/recurrence in patients withdrawing from mADM after MBCT.

Major depressive disorder is one of the most common mental disorders. Many patients with major depressive disorder experience one or more relapses or recurrences.^1^ For patients with recurrent depression, maintenance antidepressant medication (mADM) helps to reduce the risk of relapse/recurrence.^2–4^ Hence, clinical guidelines typically recommend that they should continue their medication for at least 1 or 2 years.^5,6^ Although mADM can be effective, it has several disadvantages. First, patients often experience side-effects^5^ and long-term adherence is typically low.^7^ Second, the protective effects of mADM do not persist after discontinuation whereas psychological interventions appear to have long-term beneficial effects.^8^ In addition, many patients prefer psychological treatment.^9^ Therefore, developing alternative strategies for prevention of depressive relapse/recurrence is important. Mindfulness-based cognitive therapy (MBCT) is an innovative psychological approach for relapse/recurrence prevention in recurrent depression developed by Segal, Williams & Teasdale.^10^ It is an 8-week group-based treatment that integrates elements from mindfulness-based stress reduction (MBSR)^11^ and cognitive–behavioural therapy (CBT).^12^ A meta-analysis^13^ showed that MBCT reduces the 12-month relapse/recurrence risk compared with usual care or placebo, with a relative risk reduction of 43%, in patients with a history of three or more episodes of depression. Based on this evidence, MBCT is recommended for patients in remission who are at high risk of depressive relapse/recurrence.^6,14^ In their meta-analytic paper, Piet & Hougaard^13^ also addressed the comparison between MBCT and mADM, based on two studies^15,16^ with a total of 177 participants. For patients with a history of three or more depressive episodes, MBCT with discontinuation of mADM was at least as effective as mADM alone in reducing relapse/recurrence (risk ratio (RR) = 0.80, 95% CI 0.60–1.08). This finding has recently been replicated in a large trial including 424 patients.^17^ These findings suggest that MBCT could be a viable alternative to mADM for patients who would like to withdraw from their medication. However, previous trials did not directly compare MBCT alone with the combination of MBCT and mADM. Therefore, the main aim of this multicentre, non-inferiority effectiveness trial was to examine whether patients who receive MBCT for recurrent depression in remission could safely withdraw from mADM, i.e. without increased relapse/recurrence risk, compared with the combination of these interventions. Patients were randomly allocated to MBCT followed by discontinuation of mADM or MBCT+mADM. The study had a follow-up of 15 months. Our primary hypothesis was that discontinuing mADM after MBCT would be non-inferior, i.e. would not lead to an unacceptably higher risk of relapse/recurrence, compared with the combination of MBCT+mADM. In addition, we explored whether there was a difference between the groups in terms of time to relapse/recurrence, severity of (residual) depressive symptoms during follow-up, number, duration and severity of relapse/recurrence, and quality of life.

## Method

The study design and procedures are presented in full in the study protocol^18^ (ClinicalTrials.gov: NCT00928980). They are described in brief below. Originally we intended to conduct a three-armed randomised controlled trial (RCT) of MBCT alone, mADM alone or MBCT+mADM, but this turned out to be too complicated because of strong treatment preferences expressed by patients. Therefore, we decided to conduct two parallel RCTs: an RCT comparing MBCT followed by discontinuation of mADM *v*. MBCT+mADM for patients wanting MBCT (current trial), and an RCT comparing MBCT+mADM *v*. mADM alone for patients wanting to hold on to their medication^19^ (parallel trial). Thereby we acknowledged patients' preferences while maintaining the experimental rigor of randomisation. This change has been described in the study protocol.^18^ The study was approved by the Medical Ethics Committee Arnhem-Nijmegen (nr. 2008/242) for all participating sites. After a complete description of the study, written informed consent was obtained from all participants.

### Participants

Patients were recruited in 12 secondary and tertiary psychiatric out-patient clinics across The Netherlands between September 2009 and January 2012. Patients were referred by mental healthcare professionals or recruited by advertisements in the media (television, magazines and newspapers). Inclusion criteria were a history of at least three depressive episodes according to the DSM-IV;^20^ in full or partial remission, defined as not currently meeting the DSM-IV criteria for major depressive disorder; currently treated with antidepressants for at least 6 months; 18 years of age or older; and Dutch speaking. Exclusion criteria were: bipolar disorder; any primary psychotic disorder (current and previous); clinically relevant neurological/somatic illness; current alcohol or drug dependency; high dosage of benzodiazepines (>2 mg lorazepam equivalents daily); recent electroconvulsive therapy (<3 months ago); previous MBCT and/or extensive meditation experience (for example retreats); current psychological treatment with a frequency of more than once per 3 weeks; and inability to complete interviews and self-report questionnaires.

### Procedure

Eligibility was assessed with the Structured Clinical Interview for DSM-IV (SCID) Axis 1 Disorders^21^ in a research interview performed by independent, trained research assistants. Eligible patients were randomly assigned to MBCT followed by guided discontinuation of mADM (‘MBCT+discontinuation’) or to MBCT with continuation of mADM (‘MBCT+mADM’). Randomisation was performed using a website-based application, developed specifically for this study by an independent statistician, with a minimisation procedure per research centre, stratified for full *v*. partial remission (scoring respectively ⩽11 or >11 on the Inventory of Depressive Symptomatology – Clinician rated (IDS-C)^22,23^), number of past episodes (3–4 *v*. ⩾5), prior CBT (yes/no) and gender. Allocation was performed with a 1:1 ratio. Randomisation took place after the clinical interview and participants were informed about the assigned treatment immediately by the research assistant. The research assistants conducting the assessments could not be masked to treatment group since they were also involved in the practical organisation of the trial. The baseline assessment took place after randomisation and participants started MBCT within 2 months of randomisation. The date of the first scheduled MBCT was considered the start date of the study. Follow-up assessments took place 3, 6, 9, 12 and 15 months after this start date. Participants were assessed to the end of the follow-up period irrespective of relapse/recurrence. The assessments took place between September 2009 and June 2013.

### Interventions

#### MBCT+discontinuation group

MBCT was largely based on the protocol by Segal, Williams & Teasdale^24^ with some adaptations. The intervention consisted of 8 weekly sessions of 2.5 h (instead of 2 h) and 1 day of silent practice between the sixth and seventh session (which originates from the MBSR curriculum^11^ and is suggested in the most recent version of the MBCT protocol^10^). It was delivered in groups of 8–12 participants. MBCT included formal meditation exercises, such as the body scan, sitting meditation, walking meditation and mindful movement as well as informal exercises, such as bringing present-moment awareness to everyday activities. Cognitive–behavioural techniques included education, monitoring and scheduling of activities, identification of negative automatic thoughts and devising a relapse prevention plan. Participants were encouraged to practice meditation at home for about an hour a day using CDs.

MBCT was provided in 12 different centres across The Netherlands, with a total of 19 teachers and 111 MBCT courses. Groups were mixed comprising patients from both treatment groups as well as patients not included in the trial. Videotapes of the MBCT sessions were available for 15 teachers. Two tapes per teacher were randomly selected. Teacher competency was examined by two independent expert raters using the Mindfulness-Based Interventions: Teaching Assessment Criteria comprising six domains.^25^ We report the average rating of these domains on a scale from 1 to 6 (incompetent, beginner, advanced beginner, competent, proficient, advanced).

Patients were asked and recommended to withdraw gradually from their antidepressants over a period of 5 weeks, starting after the seventh session of MBCT. This was supervised by psychiatrists. A protocol for medication tapering developed for this study by two experts in pharmacological treatment of major depressive disorder (W.A.N. and M.B.J.B.) was provided. For discontinuation we recommended a minimum of 3 and a maximum of 12 consultations during the follow-up period. Adherence to the study protocol was defined as attending four or more MBCT sessions, as in previous studies,^15,26^ and having fully discontinued mADM before the 6-month follow-up assessment (i.e. within 6 months after baseline and within approximately 3–4 months after the last MBCT session).

#### MBCT+mADM group

MBCT was provided as described above. For continuation of mADM, a minimum of one consultation with a psychiatrist was recommended. Psychiatrists were instructed to maintain or reinstate an adequate dose of antidepressants, and recommendations to manage side-effects were provided. Adherence to the study protocol was defined as attending four or more MBCT sessions and using a therapeutic dose of mADM at each follow-up contact during the observed time period (using last observation carried forward for participants who did not complete all assessments).

### Outcome measures

#### Primary outcome

The primary outcome measure was relapse/recurrence as measured with the SCID-I by trained research assistants every 3 months during the follow-up period.^18^ We purposively selected a sample of audiotaped SCID-I interviews (*n* = 35) across all study centres and across different levels of depression severity (mild, moderate or severe). These were rated by masked raters. The interrater agreement between first and second ratings was found to be substantial (kappa (κ) = 0.70, 95% CI 0.46–0.94, *P* = 0.001).^27^

#### Secondary outcomes

Time to relapse/recurrence was calculated in weeks from the start of the study until the start of the first relapse/recurrence. Patients whose follow-up data were unavailable or who did not experience a relapse/recurrence before the end of the follow-up period were treated as censored observations (with relapse/recurrence status set at 0 (‘no’) and time to event set at the number of weeks in the study with a maximum of 65 weeks). Severity of (residual) depressive symptoms was measured with the Dutch version of the IDS-C^22^ at every assessment during the 15-month follow-up period. The IDS-C has good psychometric qualities.^23,28^ In addition, number of relapses/recurrences (1 *v*. >1) and duration (in weeks) and severity (mild, moderate or severe) of the first relapse/recurrence were examined.

Quality of life was assessed at baseline, 3 and 15 months using the 26-item self-report WHOQOL short version.^29^ The WHOQOL assesses subjective quality of life in four domains: physical, psychological, social and environmental. Two questions with regard to overall perception of quality of life and health are included as well.

### Statistical analysis

For the sample size calculation we refer to the published protocol.^18^ In line with this protocol, we first report analyses based on intention-to-treat (ITT), followed by per-protocol analyses. The ITTsample included all participants as randomised; the per-protocol sample included those participants who adhered to both treatment protocols (see ‘Interventions’). For the primary outcome (relapse/recurrence) we calculated the one-sided 95% confidence interval of the difference in relapse/recurrence rates between the groups using R version 3.1.1. To conclude non-inferiority, the upper margin of the 95% confidence interval, i.e. the maximum difference between groups within this 95% confidence interval, should not exceed our non-inferiority margin of 25%. In line with regulatory guidelines on non-inferiority trials,^30,31^ this should be the case for both ITT and per-protocol, as per-protocol analysis may be more conservative in non-inferiority studies. In addition, a generalised linear model using a binomial family with an identity link was used to compare relapse/recurrence rates.

Differences in time to relapse/recurrence were analysed using a Cox regression proportional hazards model using SPSS 20.0. Regression analyses were performed with and without adjustment for depressive symptoms at baseline and number of depressive episodes in the past (Log transformed), factors known to predict relapse/recurrence.^32^ As there were no effects of research site on relapse/recurrence, we did not include this factor in our statistical models. Severity of (residual) depressive symptoms (IDS-C) during the 15-month follow-up period was analysed using a latent growth curve model in MPlus version 7 with six time points, a random intercept and a random slope for group. Participants who did not complete all assessments were included in the analyses using full information maximum likelihood estimation for missing data.

In patients who had a relapse/recurrence, number and severity were analysed using Pearson χ^2^ tests. Differences in duration of (first) relapse/recurrence were analysed with independent-samples t-tests. Quality of life was analysed with a repeated-measures ANOVA for both the observed data-set and the imputed data-set, using multiple imputation for missing data. Moderation analyses were performed using Cox regression analyses for the stratification factors (a) full *v*. partial remission, (b) number of prior depressive episodes (3 or 4 *v*. 5+), (c) prior CBT (yes *v*. no), and (d) gender. Probability values lower than 0.05 were considered significant for all analyses.

## Results

### Patient characteristics, flow and adherence

Table 1 shows the baseline demographic and clinical characteristics of the participants (MBCT+discontinuation group: *n* = 128, MBCT+mADM group: *n* = 121). There were no major imbalances between the groups. Within the MBCT+discontinuation group, patients who did not adhere to the protocol, i.e. attending fewer than four MBCT sessions and/or staying on medication, had higher levels of residual depressive symptoms at baseline (14.4, s.d. = 10.5) than those who followed the protocol (10.9, s.d. = 8.6; *t* = 2.09, *P* = 0.04, *d* = 0.37).

**Table 1:** Baseline demographic and clinical characteristics of patients with recurrent depression receiving mindfulness-based cognitive therapy (MBCT) followed by discontinuation of maintenance antidepressant medication (MBCT+discontinuation) or MBCT plus maintenance antidepressant medication (MBCT+mADM)

	MBCT+discontinuation group		MBCT+mADM group	
Variable	ITT (*n* = 128)	Per-protocol (*n* = 67)	ITT (*n* = 121)	Per-protocol (*n* = 68)
Women, *n* (%)	92 (72)	52 (78)	76 (63)	40 (59)

Educational level, *n* (%)				
Low	9(7)	6 (9)	8 (7)	5 (7)
Middle	40 (31)	19 (28)	25 (21)	13 (19)
High	73 (57)	41 (61)	81 (67)	48 (71)
Missing	6(5)	1 (2)	7 (6)	2 (3)

Marital status, *n* (%)				
Single	31 (24)	17 (25)	25 (21)	14 (21)
Married/cohabiting	72 (56)	38 (57)	69 (57)	44 (65)
Divorced/widowed	20 (16)	11 (16)	21 (17)	9 (13)
Missing	5(4)	1 (2)	6 (5)	1 (1)

Employed, *n* (%)	84 (66)	50 (75)	75 (62)	39 (57)

Remission, *n* (%)				
Full, IDS-C ⩽11	70 (55)	40 (60)	63 (52)	31 (46)
Partial, IDS-C >11	58 (45)	27 (40)	58 (48)	37 (54)

Type of mADM, *n* (%)				
Selective serotonin reuptake inhibitor	92 (72)	53 (79)	98 (81)	54 (79)
Tricyclic antidepressants	26 (20)	11 (16)	16 (13)	10 (15)
Other^a^	10 (8)	3 (5)	7 (6)	4 (6)

Previous cognitive–behavioural therapy treatment, *n* (%)	76 (59)	36 (54)	72 (60)	40 (59)

Suicide attempt (lifetime), *n* (%)	25 (20)	14 (21)	22 (18)	13 (19)

Age, years: mean (s.d.)	50.7 (10.6)	50.0 (11.3)	49.9 (10.5)	50.6 (10.7)

Baseline depression (IDS-C), mean (s.d.)	12.6 (9.6)	10.9 (8.6)	12.6 (10.5)	13.5 (10.2)

Number of previous episodes, mean (s.d.)	5.9 (5.3)	6.0 (5.3)	5.6 (4.1)	5.5 (3.5)

Age at major depressive disorder onset,^b^ mean (s.d.)	25.0 (11.7)	26.9 (12.1)	25.0 (11.8)	25.9 (12.6)

ITT, intention-to-treat; IDS-C, Inventory of Depressive Symptomatology – Clinician rated.

a. Including serotonin-noradrenaline reuptake inhibitors, monoamine oxidase inhibitors and mirtazapine.

b. Based on self-report.

**Fig. 1:**
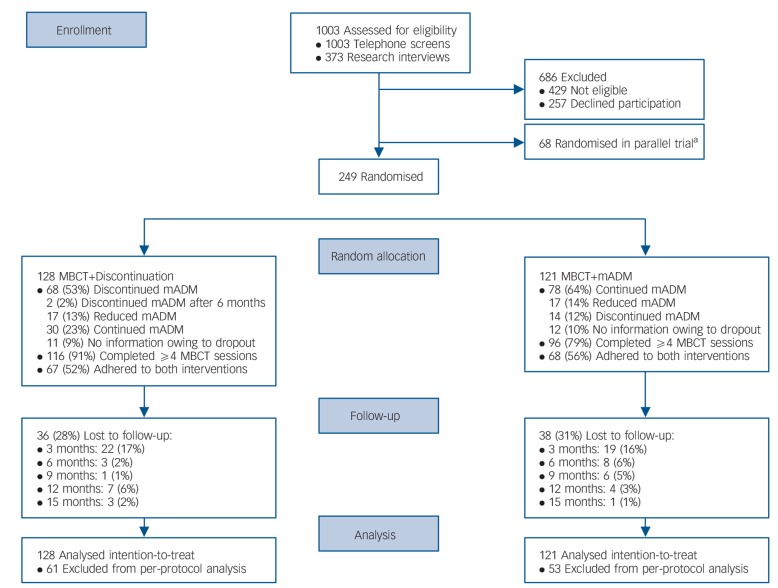
Flow of participants from screening to analysis, comparing mindfulness-based cognitive therapy (MBCT) followed by discontinuation of maintenance antidepressant medication (MBCT+discontinuation) with MBCT plus maintenance antidepressant medication (MBCT+mADM). a. Patients with a relative preference for MBCT were included in the current trial, whereas patients with a relative preference for mADM were included in a parallel trial (MBCT+mADM *v*. mADM alone).^18^

Figure 1 shows the flow of participants from screening to analysis. Follow-up assessments were incomplete for 74/249 participants (30%) of which 41/249 (16%) did not complete any follow-up assessment. Adherence to MBCT was higher in the MBCT+discontinuation group (116/128, 91%) than in the MBCT+mADM group (96/121, 79%; χ^2^ = 6.26, *P* = 0.01). The number of patients adhering to the discontinuation regimen was 68/128 (53%) and 78/121 patients (64%) adhered to the continuation regimen (χ^2^ = 3.30, *P* = 0.07). No unexpected harms were reported.

The teacher competency ratings showed that none of the teachers was incompetent, two teachers (13%) were characterised as beginners, six (40%) as advanced beginners, four (27%) as competent, three (20%) as proficient, and none as advanced. The mean teacher competency score was 3.5 (s.d. = 0.9, range 2.0–5.2). Further details will be published elsewhere.

### Primary outcome: relapse/recurrence

**Fig. 2:**
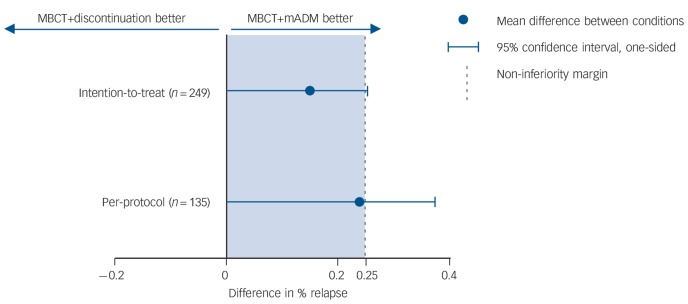
Plot showing the differences in relapse/recurrence rates and corresponding one-sided 95% confidence intervals exceeding the non-inferiority margin, for patients with recurrent depression receiving either mindfulness-based cognitive therapy (MBCT) followed by discontinuation of maintenance antidepressant medication (MBCT+discontinuation group, *n* = 128) or MBCT plus maintenance antidepressant medication (MBCT+mADM, *n* = 121).

In the ITT sample, 69/128 participants in the MBCT+discontinuation group (54%) and 47/121 in the MBCT+mADM group (39%) experienced a relapse/recurrence (RR = 1.38, 95% CI 1.05–1.83). The upper margin of the one-sided 95% confidence interval of the difference between the groups was 25.3%, which exceeded our predefined non-inferiority margin of 25% (Fig. 2). In the per-protocol sample, relapse/recurrence during the 15-month follow-up occurred in 46/67 patients (69%) randomised to MBCT+discontinuation compared with 31/68 patients (46%) randomised to MBCT+mADM (RR = 1.5, 95% CI 1.11–2.05). The upper margin of the one-sided 95% confidence interval was 36.7%, which also exceeded the non-inferiority margin. This implies that non-inferiority could not be shown. In addition, relapse/recurrence rates were significantly different between the groups for the ITT sample (Wald χ^2^ = 5.81, *P* = 0.02) and for the per-protocol sample (Wald χ^2^ = 7.76, *P* = 0.005).

To examine whether there was differential drop-out based on relapse/recurrence, we assessed whether participants who did not complete the entire follow-up period showed a higher rate of relapse/recurrence at the last observation than individuals who completed follow-up at the final assessment. In this procedure we excluded the participants who dropped out before the first follow-up assessment (*n* = 22 in the MBCT+discontination group and *n* = 19 in the MBCT+mADM group). Of all the patients with one to four follow-up assessments, 9/33 (27%) had a relapse/recurrence at the last assessment, and in the complete case group, 43/175 (25%) had a relapse/recurrence at 15-month follow-up. These proportions were comparable (χ^2^ = 0.12, *P* = 0.74).

### Secondary outcomes

**Fig. 3:**
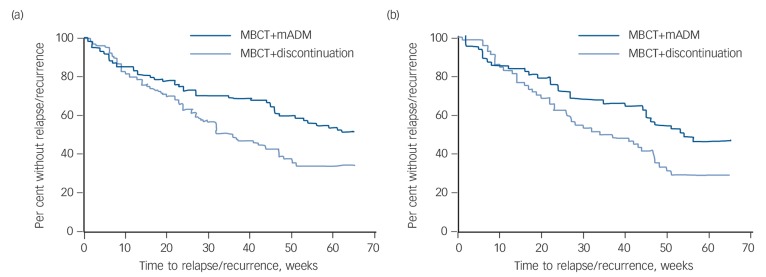
Survival curves over 15-month follow-up (65 weeks) for patients with recurrent depression receiving either mindfulness-based cognitive therapy (MBCT) followed by discontinuation of maintenance antidepressant medication (MBCT+discontinuation, *n* = 128) or MBCT plus maintenance antidepressant medication (MBCT+mADM, *n* = 121). (a) Intention-to-treat analysis, (b) per-protocol analysis.

Figure 3 shows the survival (i.e. non-relapse/recurrence) curves over the 15-month follow-up period for the ITT and per-protocol samples. The MBCT+discontinuation group was associated with an increased relapse/recurrence risk over the 15-month follow-up period (ITT: hazard ratio (HR) = 1.59, 95% CI 1.10–2.31, *P* = 0.01 and per-protocol: HR = 1.59, 95% CI 1.01–2.51, *P* = 0.05). Results were similar for analyses adjusted for baseline depression severity and number of previous episodes. Results of the moderation analyses are shown in online Table DS1. In summary, no moderation effects of full *v*. partial remission, number of prior depressive episodes (3 or 4 *v*. 5+), prior CBT or gender on the effect of group were observed.

**Fig. 4:**
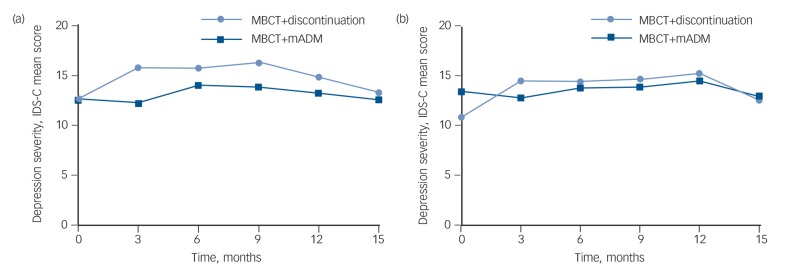
Severity of (residual) depressive symptoms over 15-month follow-up for patients with recurrent depression receiving either mindfulness-based cognitive therapy (MBCT) followed by discontinuation of maintenance antidepressant medication (MBCT+discontinuation, *n* = 128) or MBCT plus maintenance antidepressant medication (MBCT+mADM, *n* = 121). (a) Intention-to-treat analysis, (b) per-protocol analysis. Inventory of Depressive Symptomatology – Clinician rated (IDS-C) cut-off points for depression severity: 0–11 none, 12–23 mild, 24–36 moderate, 37–46 severe and 47–84 very severe.

The latent growth curve analysis for severity of (residual) depressive symptoms (IDS-C) over the 15-month follow-up period showed an acceptable model fit, χ^2^(d.f. = 20) = 39.85 (*n* = 249), *P* = 0.005, comparative fit index (CFI) = 0.95, root mean square error of approximation (RMSEA) = 0.06, standardised root mean square residual (SRMR) = 0.06. On average, patients maintained mild levels of depression throughout the study period (Fig. 4). The course of depression did not significantly vary between the groups (β = −0.05, *P* = 0.66 and β = −0.10, *P* = 0.43 for the ITT and per-protocol analyses, respectively). However, when looking at the individual follow-up assessments separately, the MBCT+discontinuation group had higher levels of depression at 3-month follow-up (15.4) than the MBCT+mADM group (12.2). This difference was small but significant (*F*(188) = 5.8, *P* = 0.02, *d* = 0.28). Depression levels did not differ between groups at the other follow-up assessments. In the per-protocol sample, there was a smaller difference at 3-month follow-up, indicated by a trend (F(117) = 3.1, *P* = 0.08, *d* = 0.20).

For those patients experiencing a relapse/recurrence, there were no differences between the groups in terms of number of relapses/recurrences (ITT: χ^2^ = 0.02, *P* = 0.89; per-protocol: χ^2^ = 1.43, *P* = 0.23), duration of (first) relapse (ITT: *t* = 0.03, *P* = 0.97; per-protocol: *t* = 0.18, *P* = 0.86) or severity of (first) relapse/recurrence (ITT: χ^2^ = 1.07, *P* = 0.59; per-protocol: χ^2^ = 3.32, *P* = 0.19). Online Table DS2 shows the results for the analyses on quality of life. In summary, there were no differences between the groups with regard to quality of life at 3-months and 15-months follow-up.

## Discussion

### Principal findings and comparison with other studies

This is the first study comparing MBCT followed by discontinuation of mADM with the combination of these interventions (MBCT+mADM). The findings of this effectiveness study reflect an increased risk of relapse/recurrence for patients withdrawing from mADM after having participated in MBCT for recurrent depression. The overall course of depression severity during the 15-month follow-up period was similar in both groups, although a small difference was observed at 3-month follow-up. At that point, parallel with the moment of discontinuation, patients in the withdrawal group had higher depression levels. In addition, this study found no differences between the groups in terms of number, duration and severity of relapse in those patients who relapsed. Moreover, quality of life did not differ between the groups.

Our results seem to be in line with evidence that combination therapy is more effective than monotherapy in the acute treatment of major depressive disorder^33^ and also for prevention of relapse/recurrence.^34^ On the other hand, earlier studies demonstrated that the efficacy of MBCT followed by withdrawal from mADM, performed in carefully controlled research circumstances, was similar to that of mADM alone.^15–17^ These findings suggest that MBCT might protect patients with recurrent depression from relapse/recurrence. In those studies, the withdrawal process took place as part of the intervention at the same time in the whole group. Under those circumstances, there may have been more emphasis on using mindfulness skills to accept the symptoms as they were and to disengage from anticipatory fears and worries. In contrast, MBCT was delivered in mixed groups in the current study. Consequently, patients might have felt more insecure, have lacked support compared with other studies and might even have been actively discouraged to pursue discontinuation by fellow-participants and/or their attending psychiatrists.

In addition, relapse/recurrence rates after discontinuation of antidepressants were somewhat higher in the current study (ITT 54% and per-protocol 69%) compared with earlier studies reporting estimates around 45% within 12–15 months.^4,15,35^ Participants in the current study may have been more at risk for relapse as about half of them were in partial (rather than full) remission and their level of baseline depressive symptoms was relatively high, for example compared with the studies by Kuyken and colleagues.^15,17^ In addition, a substantial number of the participants in this trial were reluctant to discontinue their mADM, partly based on previous unsuccessful attempts to do so, as were their attending psychiatrists. So, adherence to the discontinuation protocol was substantially lower in our study (53%) than in the studies by Kuyken and colleagues (75% and 71%).^15,17^ Patients who were not willing to withdraw may have been sensible to do so, given their higher levels of baseline depression in comparison with those who adhered to the protocol. On the other hand, previous studies have shown that discontinuation of antidepressants can be hampered by withdrawal effects^36,37^ that may be misinterpreted as signs of a possible relapse/recurrence.^38,39^

### Strengths and limitations

The great strength of the study is that MBCT was delivered in a ‘real-life’ setting, from university hospitals to community mental health centres across The Netherlands, by teachers with varying experience and competence, and in groups mixed with other types of patients. In addition to the standard dichotomous classification of relapse/recurrence we also assessed depressive symptoms over time as a continuous measure. In contrast with the higher relapse/recurrence rate, the overall course of depressive symptoms was similar between groups. This raises the question what parameter might be the better measure of the patients' suffering. Some authors have argued for the latter.^40,41^ A limitation of our approach was that the intervals between assessments were relatively long and that we did not include a self-report measure of depression.

In terms of patient selection, a limitation of the current study is that the results may apply to patients in secondary and tertiary care, but cannot be assumed to extrapolate to primary care patients or patients with less severe forms of depression. Discontinuation may be particularly unfavourable for patients who are at highest risk for relapse/recurrence because of residual symptoms and several previous episodes, as sampled in the current study.

A methodological limitation was that it was practically impossible to keep the research assistants at the different sites masked to group. However, a substantial interrater agreement (κ = 0.70) was found for the primary outcome measure (relapse/recurrence according to SCID-I) when compared with a second rater who was truly masked to group. Moreover, the relapse/recurrence rates in ITT analysis may have been biased because of incomplete follow-ups, assuming no relapse at the time of censoring. Estimates obtained from complete case analysis were higher, i.e. 61/92 (66%) relapse/recurrence in the MBCT+ discontinuation group and 40/83 (48%) in the MBCT+mADM group, and probably more realistic.

### Clinical and research implications

In summary, in routine clinical practice, patients with recurrent depression should be generally recommended to stay on medication and be informed that discontinuation of mADM may be associated with an increased risk of relapse/recurrence. This does not necessarily mean, however, that patients who do want to discontinue their mADM should not be supported to have a try or that they could not benefit from MBCT. A recommendation for future research and clinical practice would be to ensure that the clinician who supports the process of discontinuation is also trained in the mindfulness model so that the difficulties that come up can be managed within this framework. Given the many psychological factors that may influence discontinuation, placebo-controlled studies might inform us on the relative contributions of physical and psychological barriers that patients encounter. In addition, further qualitative research on the barriers and facilitators that patients experience during the discontinuation process might help us to better tailor our interventions to support them.
